# Lipid Metabolism Interplay in CRC—An Update

**DOI:** 10.3390/metabo12030213

**Published:** 2022-02-26

**Authors:** Dana Krauß, Ourania Fari, Maria Sibilia

**Affiliations:** Center for Cancer Research, Comprehensive Cancer Center, Medical University of Vienna, Borschkegasse 8a, 1090 Vienna, Austria; ourania.fari@meduniwien.ac.at

**Keywords:** colorectal cancer, lipid metabolism, metabolites, immunometabolism

## Abstract

Colorectal cancer (CRC) to date still ranks as one of the deadliest cancer entities globally, and despite recent advances, the incidence in young adolescents is dramatically increasing. Lipid metabolism has recently received increased attention as a crucial element for multiple aspects of carcinogenesis and our knowledge of the underlying mechanisms is steadily growing. However, the mechanism how fatty acid metabolism contributes to CRC is still not understood in detail. In this review, we aim to summarize our vastly growing comprehension and the accompanied complexity of cellular fatty acid metabolism in CRC by describing inputs and outputs of intracellular free fatty acid pools and how these contribute to cancer initiation, disease progression and metastasis. We highlight how different lipid pathways can contribute to the aggressiveness of tumors and affect the prognosis of patients. Furthermore, we focus on the role of lipid metabolism in cell communication and interplay within the tumor microenvironment (TME) and beyond. Understanding these interactions in depth might lead to the discovery of novel markers and new therapeutic interventions for CRC. Finally, we discuss the crucial role of fatty acid metabolism as new targetable gatekeeper in colorectal cancer.

## 1. Introduction

Combined efforts in the field of colorectal cancer (CRC) have tremendously advanced the understanding, prognosis and treatment of this deadly disease over the past decades. Main contributors for the overall improved outcome are preventive screening strategies for early detection, as colonoscopy, stool (DNA) analysis and therapeutic options through blood-based biomarkers, personalized treatments and immunotherapy [1,2,3]. Though, as of today, alarming and devastating global patterns are being recognized and show themselves in the extensively increasing number of incidences especially in young adults over the past 25 years, ranking CRC still as third (US) and fourth worldwide deadliest cancer entity [2,4,5,6,7,8]. The mortality, morbidity and early onset of CRC are expected to increase even further and main contributors to this development are not entirely understood, though hypothesized to root in changed lifestyle habits, such as smoking, diet, alcohol and obesity [9,10]. CRC development, initiation and progression are known to be influenced by various factors [11]. Apart from genetic alterations, changes in cellular metabolism are thought to even precede and trigger malignant transformation of cells [12]. 

Cellular metabolism, initially brought to attention by Otto Warburg in 1924, described that cancer cells shift their metabolism towards glycolysis and lactic acid fermentation, rather than oxidative phosphorylation to suffice their need of macromolecules and support rapid proliferation [13,14]. Over the past decades, research of cancer cell metabolism has had several renaissances and the understanding has expanded, adding cancer metabolism as an essential component to the hallmarks of cancer [15]. However, new insights have also demonstrated the reverse Warburg Effect in certain cancer entities [16]. In the recent years the focus has been on understanding glycolysis, glutaminolysis and other amino acid pathways. In comparison to proteins and nucleic acids, lipid metabolism has received less attention, but lately the reliance of cancer cells on lipid-associated pathway dysregulation has been considered yet another crucial driver of malignant transformation [17]. These complex changes in lipid metabolism where recently discussed by Molendijk et al., who describe lipid metabolism as an essential part in multiple aspects of carcinogenesis by regulating the well-described hallmarks of cancer [18].

As new analytical methods and technical advances evolved, our understanding of the lipidome is becoming more and more sophisticated [17,19,20,21]. With respect to CRC, Zaytseva et al. very recently highlighted the importance of lipid dysregulation and its implications as targets for future therapies suggesting fatty acid metabolism could be exploited as a potent vulnerability [22]. CRC shows a common altered lipid profile and many lipid-associated pathways were demonstrated to be affected, thus contributing to the development, aggressiveness and worse prognosis. Given the complexity of lipid pathway interplay in a surrounding of a many-layered heterogeneity of different cell types, the exact contribution of lipids to transformation and tumor progression are currently under investigation and many open questions remain.

This review aims to summarize the quickly growing field and update the current knowledge about lipid metabolism in CRC and how it might be exploited as a new targetable gatekeeper for cancer therapy. First, we are focusing on epithelial cancer cells and their altered sources of intracellular fatty acid pools. We outline a functional and metabolic pathway-centric description, integrate underlying molecular concepts, leaving out driver mutations, transcriptional regulation (comprehensively reviewed elsewhere [23]) and briefly cover highly complex interactions of microbiota with the intestinal epithelium (recently reviewed here [24,25,26]). Additionally, we highlight the role of lipid metabolism in cell communication and crosstalk within the tumor microenvironment.

## 2. Lipid Biology in CRC

Lipids are a class of hydrophobic or amphipathic molecules estimated to ~180.000 different species [27], categorized into eight main groups: fatty acids (FA), glycerolipids, glycerophospholipids, sphingolipids, sterols, prenols, saccharaolipids and polyketides [28]. FAs will be the major focus of this review. They are the main structural building blocks for complex lipids, providing a carboxylic acid group and hydrocarbon chains in varying length and (un-)saturation, thus defining their bio-physical properties. The number of aliphatic chains groups them into short-chain fatty acids (SCFA, 1–6), medium-chain fatty acids (MCFA, 7–12), long-chain fatty acids (LCFA, 13–20) or very long-chain fatty acids (VLCFA, >20). Based on presence and addition of double bonds, FAs can be saturated fatty acids (SFA) or unsaturated fatty acids (UFA). UFA include monounsaturated fatty acids (MUFA, carrying one double bond) and polyunsaturated fatty acids (PUFA, two or more double bonds) [29]. Lipids have diverse roles in the entire human body but are molecularly essentially involved in providing cellular structure, energy sources and signaling events. They are the major building blocks for cellular membranes and depending on the composition alter and modulate its fluidity. Aside from providing an energy reservoir, lipids have important cell signaling functions by acting as secondary messengers [19].

**Figure 1:**
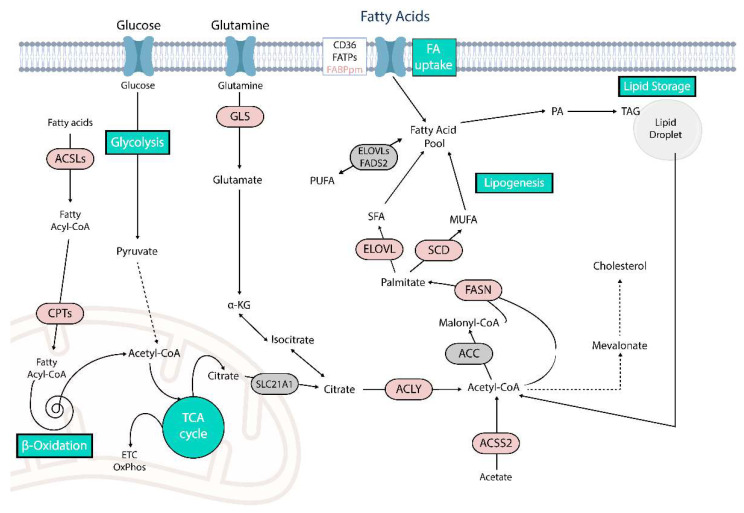
Simplified overview of fatty acid metabolic reprogramming in CRC. Schematic representation of main fatty acid metabolism associated processes and enzymes emphasized in this review. Enzymes shown to be upregulated in CRC are highlighted in red. ACC, acetyl-CoA-carboxylase; ACLY, ATP-citrate lyase; ACSS, acyl coenzyme A synthetase; aKG, alpha-ketoglutarate; ACSL, acyl-CoA synthetase long-chain family; CPT, carnitine palmitoyltransferase; ELOVL, elongation of very-long-chain fatty acids protein; ETC, electron transport chain; FA, fatty acid; FABP, fatty acid binding protein; FADS, fatty acid desaturase; FASN, fatty acid synthase; FATP, fatty acid transport protein; GLS, glutaminase; MUFA, monosaturated fatty acid; PA, phosphatidic acid; PUFA, polyunsaturated fatty acid; SCD, stearoyl-CoA desaturase; SFA, saturated fatty acid; TAG, triacylglycerols.

The effector roles and molecular properties are what link lipids and their metabolism tightly to their intrinsic features and explain their contribution to cancer development and progression. Understanding the precise connections is important to comprehend how FA sources and provision are changed, rewired and what specific determinants are affected in CRC. It is acknowledged that CRC cells show an abnormal lipid metabolism, primarily manifested by upregulated lipogenesis, involving *de novo* fatty acid synthesis and triglyceride synthesis, increased uptake and abundance of lipids and in general reliance on FAs [30]. Phenotypically, this is evidenced by an shifts in total lipid content in tumor compared to healthy tissues, altered triacylglycerol and cholesterol storage in lipid droplets (LDs) [31,32] and differential expression of genes encoding for lipogenic related enzymes [33] (affected pathways summarized in Figure 1). As a consequence, abnormal lipid metabolism of CRC cells affects numerous processes such as apoptosis, autophagy, necrosis, proliferation, differentiation, growth, plasticity and thereby drives tumor initiation and progression which are significant for the advancement and poor prognosis of CRC [12,30,34,35,36].

### 2.1. Altered Fatty Acid Sources in CRC

Highly proliferative CRC cells have a high demand and need for new building blocks, of which a major fraction contributes to building cellular membranes. Membrane lipids are composed of phospholipids such as phosphoglycerides and sphingolipids, glycolipids and cholesterol. Composition and modulation of cell membrane fluidity is important for protein function and dynamics, mediating organization of local microdomains. Therefore shifts in FA sources indirectly impact membrane function [37]. Relative reduction of PUFA and increased SFA over UFA incorporation are thought to protect from lipid peroxidation-mediated cell death and metabolic stress, by neutralizing reactive oxygen species (ROS) and thus increasing cell survival, tumor progression and metastasis [19,38,39]. *De novo* fatty acid synthesis in CRC was shown to increase lipid raft formation within cellular membranes and thereby modulate accessibility for signal transduction proteins and additionally, tumor tissues showed distinct enriched profiles of highly complex lipid species [38,40,41,42].

Somatic cells compared to tumor cells normally take up extracellular, circulating FAs from dietary fats whereas lipid biosynthesis is usually restricted to hepatocytes, adipose tissue and mammary glands [43,44]. Cancer cells and specifically CRC cells were shown to change their source of lipids on the one hand by increasing extracellular uptake and on the other hand by reactivating *de novo* fatty acid synthesis regardless of extracellular lipid availability, providing them with metabolic flexibility and alternative sources of energy [12,42,45]. One source for scavenging extracellular lipids is adipocyte or hepatocyte-derived, plasma albumin-bound free-fatty acids. Especially, the essential fatty acids α-linolenic acid (ALA, 18:3 n-3) and linoleic acid (LA, 18:2 n-6) cannot be synthesized *de novo* by humans and other mammals and need to be taken up with the diet [46,47,48]. Other sources of fatty acids are derived from lipoproteins triacylglycerols, fatty acid ester or glycerophospholipids. Additionally, stromal sources as cancer associated fibroblast where shown to provide lipid containing exosomes [49]. Transport of local lipid supplies are mediated by different mechanisms. Extracellular lipolysis can liberate free fatty acids from lipoproteins or entire particles are endocytosed and further enter endosomal-lysosomal pathway. Extracellular free fatty acids can be transported across the membrane via different membrane associated proteins that are further described in more detail [45,50].

Membrane-associated proteins that have been described to mediate fatty acid uptake include fatty acid transport proteins (FATP), fatty acid binding proteins (FABP) and the best characterized scavenger receptor, CD36, also known as fatty acid translocase (FAT). A body of knowledge and evidence has been collected for CD36, indicating a critical role in many entities, as CRC, gastric, cervical or ovarian cancer, impacting an array of signaling pathways, but the overall conclusion concerning the role of CD36 remains inconsistent [36]. Upregulation on cancer cells was associated with poor prognosis, increased storage of FAs in LDs and activation resulted in proliferation inhibition and induced apoptosis in CRC [50,51]. Metastatic tissue shows CD36 upregulation in comparison to primary tumor tissue, thus showing a differential role that is tissue and tumor stage dependent [36]. High fat diet has been shown to boost the metastatic potential in a CD36- dependent manner, while inhibition of CD36 resulted in impaired metastasis in melanoma and breast cancer derived tumors [52,53]. Drury et al. showed a compensatory upregulation of CD36 after inhibition of fatty acid synthase (FASN) with the novel inhibitor TVB-3664 and demonstrated a synergistic effect on cell proliferation after CD36 inhibition [54]. Mechanistically, they very recently linked the observed effect to matrix metalloproteinase 29 upregulation by employing in vivo tail vein and cecum injection models [55]. However, other studies showed only minor effects after CD36 knock-out, thus the employment of CD36 as a biomarker and its translation into clinical practice is still under debate [56].

Another membrane bound protein FATPs, also described as SLC27A family membrane proteins, transport exogenous LCFA. In melanoma, FATP1 increases FA uptake, growth and invasion and specifically FATP2 increases proliferation and therapy resistance [57]. FABPs regulate FA metabolism, trafficking and are believed to act as mediators of tumorigenesis [58]. Using the APCMin model of CRC, knockout of the FABP1 isoform resulted in decreased number of adenomas [59]. High expression of FABP5 was associated with cell growth and invasion demonstrated in in vitro models [60]. FABP6 shows higher expression in sessile serrated adenomas/polyps compared to normal colonic tissue and increases intracellular bile acid transport in the ileal epithelium. Additionally, higher expression in primary tumor tissue compared to lymph node metastasis was observed [58,61]. Increased fatty acid transport by overexpressed FABP4 promotes migration, invasion and metastasis as demonstrated in patient tissue, in in vivo and in vitro models of CRC and enhances lipid metabolism through AKT pathways activation [45,62].

### 2.2. De Novo Fatty Acid Synthesis in CRC

As outlined, FAs can either be derived from extracellular sources or generated intracellularly from carbohydrate precursors. Contrary to normal cells, CRC cells feed the major fraction of their FA pool by reactivation of *de novo* lipogenesis [63]. The starting point of fatty acid synthesis is cytosolic acetyl-CoA and major subsequent steps include conversion of acetyl-CoA to malonyl-CoA, condensation and eventual elongation and desaturation. The main precursors for cytosolic acetyl-CoA are either derived from glycolytic flux or other anaplerotic sources as e.g., glutamine feeding TCA derived citrate. TCA-cycle/mitochondrial derived citrate is exported through the SLC25A1 transporter into the cytosol where the initial step of fatty acid synthesis takes place. In this rate-controlling step, ATP-citrate lyase (ACLY) converts citrate to acetyl-CoA and oxaloacetate, thereby coupling carbohydrate metabolism with fatty acid biosynthesis. High levels of ACLY in CRC cells were shown to promote metastasis and resistance to chemotherapy, among others through interaction with β-catenin 1 [64], acetylation and AKT phosphorylation events, thus posing as one key player in lipid-associated rewiring [65,66].

A different source of cytosolic/extramitochondrial acetyl-CoA under low glucose can be provided by acetate via acetyl-CoA synthetase (ACSS) family members, which are the only enzymes using acetate as substrate to produce acetyl-CoA by ATP-dependent ligation to CoA. Acetate has many sources and can be derived extracellularly or generated endogenously, thus bypassing the requirement for citrate, directly contributing to acetyl-CoA [46,67]. Particularly CRC cells are exposed to high local acetate concentration and hypoxic CRC cells were shown to increase acetate uptake through activation of ACSS2 and endogenous generation via histone deacetylases that remove acetyl from histones [67,68]. ACSS2 exists in nuclear and cytosolic isoforms and has been shown to promote cellular proliferation through enabling fatty acid synthesis from acetate [67].

Further, acetyl-CoA can be activated to malonyl-CoA through acetyl-CoA carboxylases (ACC1/2), also known as ACACA/ACACB. This irreversible carboxylation step of *de novo* lipogenesis is regulated by phosphorylation and allosteric regulation as feedback control for example by high concentrations of palmitoyl-CoA. It couples mitochondrial fatty acid synthesis with β-oxidation through the activity of carnitine palmitoyltransferases (CPTs). Inhibition of ACC was shown to be toxic and to induce dose-dependent apoptosis in CRC cells [69,70]. Subsequent condensation of one acetyl-CoA with seven malonyl-CoA creates the C16:0 SFA palmitate. FASN is the key enzyme mediating this energetically expensive process consuming seven ATPs and 14 NADPHs. Finally, palmitate is substrate for various elongation and desaturation steps, creating a variety of different FAs. 

A plethora of knowledge about consequences in FASN alteration and activity in CRC lipid metabolism has been gathered [71]. Aberrant crypt foci of early-stage neoplasms from familial adenomatous polyposis tissue with sporadic CRC, but also advanced and metastatic tissues show increased expression of this lipogenic enzyme. Specifically, upregulation was detected in whole tissue and purified EPCAM-positive cells, which coincided with increased proliferation and induction of CD44 expression, promoting invasion and migration [72,73,74,75]. High FASN activity promotes cellular respiration by enhancing glycolysis, mitochondrial respiration and β-oxidation of endogenous lipids [12,33] and induces increased saturation of membrane lipids, making cells less susceptible for lipid-peroxidation by radicals and inhibiting chemotherapy penetration [76]. Together, the described properties correlate with metastasis and angiogenesis, and therefore worsen outcome and prognosis. Mechanistic studies have linked FASN to various oncogenic pathways [71,77] and specifically growth factor receptor signaling pathways are shown to increase FASN transcription. For example, activated epidermal growth factor receptor (EGFR) was shown to upregulate FASN in pancreatic ductal carcinoma and reciprocally, silencing or inhibition with small molecule inhibitor Erlotinib abolished FASN upregulation [78]. Many other inhibitor and knock down studies in various in vivo and in vitro CRC models have shown antitumor effects, reduction of invasion and metastasis [71]. Mozolwsky et al. recently collected knowledge about FAS and oxidation inhibitors in CRC treatment, summarizing FASN inhibitors in detail [79]. Promising therapeutic results seem to be rooted in distinct FASN tissue distribution and activity between normal and cancer tissue, suggesting FASN as a potential biomarker and advancing FASN as target of currently ongoing clinical trials [33,79,80,81].

Subsequently, FASN generated SFA palmitate can be modified through elongation and desaturation. *De novo* MUFA generation through fatty acid desaturation is mediated by the principal, best characterized stearoyl-CoA desaturase 1 (SCD1). This is the key rate-limiting step for MUFA synthesis from SFAs creating a double-bond in Δ9 of stearic (C18:0) or to lesser extent of palmitic acid (C16:0). Oleic acid (C18:1) is one of the most abundant MUFAs. Desaturation is chemo-, stereo- and substrate selective and consumes NADPH and molecular oxygen generating the cellular pool of unsaturated FAs especially under lipid deprivation. Generally, SCD1 was shown to drive tumor proliferation and invasiveness through increased lipogenesis and misbalance of fine-tuned MUFA/SFA ratios, advancing the development of many chemical compounds targeting this desaturase. The role of SCD1 in CRC, however, still is not entirely understood and Piccinin et al. have recently summarized in detail the essentiality of SCD1 in CRC development, manifested by altered activity in tumors compared to healthy tissue that correlates with promotion of epithelial-mesenchymal transition [82,83].

PUFA generation is important for eicosanoid and prostaglandins production that regulate immune and inflammatory response. Fatty acyl desaturases (FADS1-3) mediate PUFA generation from the essential fatty acids LA and ALA and the degree but also position of saturation determines the susceptibility of FAs to oxidation making PUFAs more prone to oxidation [48,63]. FADS2 is often upregulated in tumors that are insensitive to SCD inhibition, thereby FADS2 maintains intracellular MUFA levels [84]. Thus, avoiding accumulation of SFAs and an imbalanced MUFA/PUFA ratio, which is advantageous to evade peroxidation and ferroptosis. Consistent with this function, increased FADS2 expression in CRC was shown to promote cancer proliferation and tumor growth [85,86]. The ratio of n-3 and n-6 PUFAs play opposite effects on inflammation and were also shown to be altered in CRC. Zhang et al. showed an increased n-6/n-3 PUFA ratio in cancer versus adjacent tissue [87], which was also observed in a later study [88]. Another study however demonstrated an increase in n-3 over n-6 PUFA ratio comparing CRC versus healthy tissue [89]. A recent detailed review elaborates on this controversy [90]. Elongation of FAs is mediated by enzymes of the FA elongase family (ELOVLs), which add two carbons from malonyl-CoA during each step, creating a multitude of very complex lipids with different specificity of the different members towards either SFAs, MUFAs or PUFAs [91]. Generally, cancer cells enhance elongation of extra- and endogenously derived FAs and specifically colorectal tumors show higher levels of specific elongated lipids and high transcript levels of ELOVL2 and ELOVL5 enzymes [19,45,89].

## 3. Fatty Acid Storage and Degradation

Storing major fractions of scavenged and *de novo* synthetized lipids intracellularly can mediate multiple advantages for tumors. LDs act as cytoplasmic organelles for storage and reservoirs for cholesterol and acylglycerols, mainly composed of triacylglycerols (TAGs) and cholesteryl esters (CE) [32,92]. A clear picture of lipid deposition in CRC cells is yet to be drawn and remains controversial. Some publications report extensive accumulation of LDs and utilization of stored lipids as source of ATP and NADPH for metabolic stressful situations and to maintain lipid homeostasis. Differentiation state, stem cell markers and Wnt activity were shown to positively correlate with high LD content in CRC stem cells [93]. The abundance of stored lipids mediates aggressiveness, chemoresistance and therapy relapse [32]. Additionally, increased TAG and CE contents within LDs show distinct saturation indices, such as increased saturation of TAG compared to CE, associated to metastasis derived cells [30,94]. One report assessing matched biopsies showed no alterations in LDs [95] and yet other reports demonstrated decreased levels of triacylglycerol contents in tumor compared to paired adjacent tissue [89]. In turn they observed increased cell membrane components, namely phospho- and sphingolipids and argue that the conducted study analyzed specific lipids in much more detail, including mono-, diacylglycerols, lysophospholipids and ceramides and suggesting observed alterations of specific lipids depends on their role in either energy generation or cell membrane contribution [42]. A recent study also demonstrated a net decrease of TG in tumors, while showing altered composition inside LDs, evidenced by VLCFA increase compared to LCFA [96]. Additionally, TGs during CRC progression was shown to be higher in early stage in one study [97] and enriched in T3 [98].

The balance between *de novo* fatty acid synthesis and β-oxidation is tightly regulated and therefore FA degradation is likewise essential for homeostasis. FAs are the preferred substrate for energy storage and their oxidation yields twice as much energy as that of carbohydrates. VLCFA require initial shortening in peroxisomes whereas FAs, from, for example, stored TAG, first need activation before they can enter mitochondrial β-oxidation, mediated by the carnitine carrier system. CPT1 on the outer mitochondrial membrane is the rate-limiting enzyme in fatty acid oxidation (FAO), activating acyl-CoA by conjugating it to carnitine. Allosteric inhibition of CPT1 by malonyl-CoA, an intermediate of fatty acid synthesis, acts as an important regulator by preventing the futile cycling between β-oxidation and synthesis, making them mutually exclusive. Carnitine-acylcarnitine-transporter (CACT or SLC25A20) then translocates to the inner mitochondrial membrane where CPT2 converts acyl-carnitine back to acyl-CoA and carnitine. During each stepwise shortening iteration of a given FA, a two-carbon acetyl-CoA unit is generated, entering the TCA cycle yielding a total of 130 ATP of the C16 fatty acid palmitate and essential NADH. Specifically, non-glycolytic tumors use FAO as their main bioenergetic pathway [99]. CRC cells show increased expression of CPT1A, which is promoting metastasis by inhibiting anoikis and knockdown or targeting CPT1A using etomoxir, showed to be effective to blunt FAO [19,100].

## 4. Colorectal Cancer Lipidome

Identification of easy to stratify biomarkers has been an attractive field of research. Great effort of many studies tried to dissect the complex lipid profile of tissue and serum samples and ongoing debate revolves around a specific CRC lipidome that would have a clinical role for the disease. Initial studies detected enhanced VLCFA and lower LCFA in serum of CRC patients. Increased elongation of saturated and monosaturated VLCFA by ELOVLs were discussed to enable this observation [30,101]. LC-MS-based serum profiles showed saturated TAG as the main perturbed lipids in CRC progression and the authors pointed towards LD accumulation as the origin [102]. Another group observed decreased levels of lyso-lipids, glycophosphocholines and acylcarnitines serum concentrations in CRC patients [103]. Very recently, Ecker et al. additionally identified a robust TG lipidomic tissue signature that could discriminate patients and was proposed as prognostic identifier. The authors performed quantitative lipidomic analysis of matched tumor samples and described altered glycerol-, glycerophospho and sphingolipid profiles. Glycerol and sphingolipids most robustly discriminated across a heterogenous set of patients with mixed mismatch repair-proficient and -deficient status, oncogenic mutations (KRAS/BRAF) or grading. Observed alterations are accompanied by elevated transcripts of lipogenic enzymes as FASN and FADS2 and the described signature could even be recapitulated in an APC mouse model [73].

## 5. Lipid Metabolism in the Tumor Microenvironment

It is now well accepted that tumor cells are greatly influenced by the surrounding tissue in which they reside. CRC consists of a complex tumor microenvironment (TME) that influences the progression of the disease. Immune cells, adipocytes, cancer-associated fibroblasts, endothelial cells, pericytes and extracellular matrix are some of the components commonly found in the TME of CRC. The various cell types normally communicate and build a delicate balance, which is commonly found disturbed in CRC, as summarized by several reviews [12,35,36]. 

Interactions between TME components and cancer cells, form a dynamic network that ultimately controls proliferation, invasion and metastasis of tumors. Metabolic adaptations and reprogramming occur within different cell types and have been the center of interest in recent years. The crosstalk between TME components leads to a complex interplay that is marked by crucial events. Firstly, cancer cells require a lot of energy production and are metabolically active, therefore they compete with their neighboring cells for nutrient availability. Nutrient depletion often drives other cells to switch to different metabolic programs or impedes their normal regulation. On the other hand, secreted metabolites in the TME can influence the function and phenotype of surrounding cell types, resulting in reduced proliferation or activation [104,105]. These events contribute to cancer progression, as they can support immunosuppression, allowing tumors to escape immunosurveillance [106]. Moreover, metabolic interactions can enhance tumor invasion and metastasis. Lipid metabolism has a central role in this interplay and has been in the spotlight as it could represent a promising pathway for pharmacological intervention and combinational therapies [46]. 

### 5.1. Immune Cells

The importance of lipid anabolic and catabolic processes has been demonstrated through the years for a variety of cell types. Among them, immune cells have been extensively studied as they display different lipid metabolic profiles, according to their functional state. Several recent publications have elegantly linked classical immune phenotypes of both innate and adaptive immunity to their metabolic state. For example, several signals, such as lipopolysaccharide (LPS) can polarize macrophages into a pro-inflammatory state, during which they upregulate fatty acid synthesis, while interleukin-4 (IL-4) polarized macrophages are characterized by increased FAO [107,108]. Dendritic cells also upregulate *de novo* fatty acid synthesis upon LPS stimulation, leading to increased lipid storage in lipid bodies, which is necessary for cross-presentation of phagocytozed antigens to CD8+ T cells [109,110]. Lymphocytes also display metabolic shifts according to their functional state. Naïve T cells normally perform minimal biosynthesis, while after their activation their fatty acid synthesis rate is increased [104,111]. On the other hand, tissue resident memory T cells (T_RM_) and T regulatory cells (Tregs) exhibit a preference for uptake of exogenous FAs, which then are oxidized in mitochondria to generate sufficient energy. Impairment of basic lipid uptake or further catabolic processes can impede Treg differentiation and survival or even lead to a shift towards other T cell phenotypes such us T helper 17 cells (Th17) [112,113,114]. 

In the context of tumors, immune cells have similar metabolic requirements with their physiological states, which can influence their function. Several different studies have shown the importance of metabolic shifts in immune cells in tumors. Upregulation of the FATP2 contributed to the suppressive phenotype of polymorphonuclear myeloid-derived suppressor cells (PMN-MDSCs), through a mechanism that involved uptake of arachidonic acid and synthesis of prostaglandin E_2_ [115]. Oleate, an UFA, could polarize bone-marrow derived macrophages to a suppressive phenotype, through accumulation of LDs and induction of FAO. Disruption of the LD formation was able to disrupt in vitro polarization of macrophages and the growth of subcutaneous CRC models [116]. Another study demonstrated that in several cancer types including CRC, tumor associated macrophages (TAMs) expressed elevated levels of the scavenger receptor CD36, accumulated lipids and utilized FAs for energy production. High FAO led to the production of ROS, leading to STAT6 activation and transcription of genes that regulate TAM generation and function [117]. Natural killer cells (NKs) from post operated CRC patients also exhibited elevated levels of CD36 and lipid accumulation, while they produced lower levels of granzyme B and perforin. The same study showed that granulocytic MDSCs from mice undergoing surgery enhanced the expression of scavenger receptors on NK cells [118]. Moreover, intracellular accumulation of oxidized triglycerides and cholesterol esters, could impede cross-presentation in dendritic cells by reducing the presence of peptide-major histocompatibility I complexes on their surface, leading to decreased proliferation of T cells [119]. Sphingolipids are also important regulators of immune responses, as loss of acid ceramidase, an enzyme important for their production, was shown to be necessary for the expansion of MDSCs in models of colitis associated CRC [120].

As already mentioned, immune cells face competitive conditions within the TME, as tumor cells upregulate metabolic pathways leading to increased biosynthesis and energy production. This often leads to deprivation of several crucial metabolites from the microenvironment, which immune cells need to fulfill their functions [104]. In mouse CRC, it has been shown that a high fat diet leads to an increased uptake of FAs from tumor cells, resulting in an altered lipid partitioning in tumors and impaired CD8 T cell infiltration and activation. Blocking of the metabolic reprogramming specifically in tumor cells, improved anti-tumor immunity [121]. In another study, deletion of the free fatty acid receptor-2 (FFAR-2) from dendritic cells, promoted their activation and expression of interleukin-27 (IL-27). Subsequently, this led to impaired mucosal barrier integrity, exhausted CD8 T cells and higher numbers of tumors [122]. In addition, in a model of colitis induced CRC, it was demonstrated that dietary supplementation of conjugated linoleic acid (CLA), had a pro-tumorigenic effect, resulting in transforming growth factor-β (TGF-β) production by macrophages and T cells, an effect that was mediated via activation of peroxisome proliferator-activated receptor-γ (PPAR-γ) [123]. In summary, these studies demonstrate how the interplay among cancer and immune cells can lead to metabolic rewiring for both sides, affecting the progression of CRC. We have also previously shown the importance of the communication between epithelial and myeloid cells expressing EGFR in the progression of colorectal cancer, which unpublished data imply that it is based on metabolic interactions [124].

### 5.2. Adipocytes

Adipocytes are commonly found within colon tissue. They produce and store lipids and they also interact with cancer cells, affecting their metabolism and growth. Colon tumors often invade into surrounding adipose tissue and build close contact with neighboring adipocytes. Exposure of cancer cells to adipocytes, led to upregulation of CPT1A and FAO in tumor cells, which in turn promoted Wnt signaling and cancer stem cell properties [125]. Moreover, primary adipocytes and preadipocytes induced the proliferation of colon cancer cell lines in in vitro co-culture systems [126]. Mechanistically, adipocytes supported cancer cells to survive nutrient deprivation by providing FAs, which subsequently were oxidized in mitochondria. The presence of adipocytes or FAs also promoted autophagy in tumor cells, as a result of activated 5′AMP-activated protein kinase (AMPK) signaling, which contributed to the growth supporting effect of adipocytes [127]. Adipocytes were also shown to support ovarian cancer progression and metastasis by providing FA and the expression of CD36 on cancer cells [128].

Systemic effects of lipid metabolism and adipocytes receive a lot of focus in cancer biology, as cancer patients often experience weight reduction, mostly reflected as fat and muscle loss, a condition termed cachexia [129]. Cancer cachexia is now viewed as a metabolic disorder, and is common among CRC patients, while it is considered a major cause of cancer related death [130,131]. Dietary habits of CRC patients are often considered responsible for shifts in metabolic profiles and even disease progression, while anorexia is considered only partly a cause for metabolic shifts and undernourishment [132,133]. Beside low food intake, adipose tissue dysfunction is a major player in cancer cachexia, during which white adipose tissue (WAT) is undergoing a browning process marked by increased lipid mobilization and energy expenditure [134]. Exosomal miR-146-5p derived from CRC cells could induce WAT browning and lipolysis, through repression of homeodomain-containing gene C10 (HOXC10), illustrating the importance of cell crosstalk in the tumor microenvironment [135]. Interestingly, in a pre-clinical model of CRC, it was shown that chemotherapy treatment decreased the size of adipocytes and also the expression of proteins involved in ATP production, β-oxidation and lipogenesis [136]. Although not fully understood yet, cancer cachexia now receives a lot of attention as an example that links local lipid metabolic events with systemic imbalance.

### 5.3. Cancer Associated Fibroblasts

Fibroblasts are found in connective tissue and produce extracellular matrix components including collagen, which are necessary for a structured stroma. Under pathological conditions, such as cancer, secreted factors from tumor cells can activate them and influence their function. Cancer Associated Fibroblasts (CAFs) are characterized by great heterogeneity and display different phenotypes among the variety of cancer types [137]. Metabolic stress can reprogram CAFs to create a nutrient rich environment to support tumor growth [138]. Breast cancer cells could induce the production of ROS in CAFs, leading to autophagy and lysosomal degradation of caveolin-1, which in turn could induce mitochondrial dysfunction in adjacent fibroblasts [139,140]. Moreover, it was demonstrated that in breast cancer, mitochondrial uncoupling proteins (UCPs) induced mitochondrial dysfunction in fibroblasts and generation of FAs and ketone bodies, which promoted tumor growth through β-oxidation [141]. Another study recently showed that reprogramming of lipid metabolism and increased expression of the FASN enzyme in CAFs, leads to lipid production and promotes migration of colorectal adenocarcinoma cells. Knockdown of FASN in CAFs or blocking of CD36 in vivo with a monoclonal antibody abolished the mobility of CRC cells [142]. 

### 5.4. Endothelial Cells

Lipid metabolism in endothelial cells (ECs) is not yet substantially characterized, although it has been shown to have an important role in several pathological conditions, such as atherosclerosis. For example, oxidized low density lipoprotein (oxLDL) and thrombospondin-1 (TSP-1) have been long known to act as signals for CD36 expressed by macrophages and endothelial cells, resulting in an inflammatory state and the development of atherosclerotic plaques [143,144]. ECs were shown to transport lipids to other cell types and CD36 was important for this process as its deletion led to deregulated FA uptake by parenchymal cells [145]. Although ECs do not proliferate under physiological conditions, during neovascularization in tumors, they switch to a proliferative and migratory state, for which metabolic adaptations are required [137]. ECs increase their glycolytic flux during this process, however glucose restriction resulted in a shift towards FAO for nucleotide synthesis, which was shown to be mediated by CPT-1 [146]. During migration, ECs were shown to synthesize lipids and ACC inhibition led to an altered membrane composition and a reduced migratory capacity [147]. In addition, cholesterol levels also seem to play a role in angiogenesis as activation of endothelial liver X receptors (LXRs), which are sensors of cholesterol, resulted in reduced vascular endothelial growth factor receptor-2 (VEGFR-2) compartmentalization and signaling. LXR agonists decreased angiogenesis and tumor size of lung Lewis carcinoma grafts [148]. In colon cancer, attenuation of lipogenesis though PI3K inhibitors could impede angiogenesis in vitro and in vivo [149].

### 5.5. Contribution of Gut Microbiota

Over the past decade, high-throughput and large-scale metagenomics studies have tremendously accelerated microbiome research [150]. Starting out, identification and annotation of individual microbial genomes was the primary focus which currently shifts to understanding molecular mechanisms underlying symbiotic cohabitation of the host and the microbiome, consisting of bacteria, viruses, fungi and protists. Bacteria benefit from space to colonize and sources of nutrition [36]. Host advantages however are not as clearly understood and include a multitude of aspects ranging from modulation of the gut mucosal immune system, supply of certain vitamins, promoting water and electrolyte absorption and maintaining energy homeostasis among many other aspects [151]. The relationship between bacteria and metabolites is a delicate interplay that enables the function of colonic epithelia and immune cells and protects against inflammation or carcinogenesis under normal physiological conditions [25]. Thus, dysbiosis and the resulting shifts in metabolites from gut microbiota are shown to be involved in the development, progression and metastasis of CRC [12,152]. As microbiota play an important role in the TME, we shortly want to highlight described lipid associated metabolites that are involved in the intricate communication between microbiota and the host. 

The SCFA butyrate is a fermentation product of carbohydrates, produced by certain species, as *Bacillotes* [153]. Butyrate’s beneficial effects are known since decades and especially its paradoxical effect on inhibiting growth of CRC cells but simultaneously promoting proliferation of normal colonic epithelium [151,154]. Mechanistically, the dual effect of butyrate is rooted in different metabolic profiles of healthy colonocytes and CRC cells. Colonocytes readily metabolize butyrate through β-oxidation. Cancer cells, however, show a glycolytic phenotype and therefore butyrate remains available, which accumulates and finally acts as a histone deacetylase inhibitor [25]. CRC patients were shown to have reduced SCFA stool levels and less of butyrate producing bacteria. Additionally, cell surface receptor for SCFA as G protein coupled receptor 109A (GPR109A) or G protein coupled receptor 43 GPR43 show altered expression [155]. Butyrate was recently shown to enhance the efficacy to radiotherapy in a patient derived organoid model [156]. Additionally, bacteria secreted SCFAs demonstrated modulation of immune responses. *Fusobacterium nucleatum* could drive a pro-inflammatory intestinal microenviroment through the SCFA receptor FFAR2, which drives a dependent modulation of IL-17 expression [157]. SCFAs have also been shown to enhance the cytotoxic activity and the memory potential of CD8 T cells. This was accomplished through butyrate mediated preferential fueling of oxidative phosphorylation through sustained glutamine utilization and fatty acid catabolism [158]. Specifically, they can promote the memory potential of antigen-activated CD8+ T cells, while dietary fibers can shape monocytes and CD8+ T cell metabolism [159]. Moreover, the commensal metabolite butyrate and niacin induced IL-18 in colon and IL-10 in antigen presenting cells. The receptor of butyrate and niacin can promote anti-inflammatory effects, against colon inflammation and colorectal cancer [155].

Bile acids and their derivatives pose another important group of lipid metabolites modified by gut microbiota [151]. Small portions of primary bile acids as cholic or chenodeoxycholic acid that escape reabsorption, enter the colon and certain microbiota mediate malignant transformation to secondary biles as deoxycholic acid (DCA) and lithocholic acid. DCA was shown to activate the EGFR pathway and downstream activator protein 1 response induced cell proliferation [160]. In APCMin models, DCA promotes pathogenesis as evidenced by increased tumor number and volume, impaired barrier function and inflammation [161]. Interested readers are referred to a detailed, recent overview by Zhang et al. of microbiota-derived metabolites and their mechanistic action [24].

## 6. Conclusions

CRC is a complex and heterogeneous pathology, marked by genetic alterations, dysregulated signaling pathways and metabolic adaptations. Although mortality of CRC decreased over the past decade, it is still an immense health burden, especially as incidences in younger patients have increased, with limited understanding for underlying causes. Disturbed lipid metabolism is now a well-accepted characteristic of several cancer entities, the major contributor being improved techniques in the field. Specifically, lipidomic coverage, high-throughput methods as chemical imaging, functional genomics and spatiotemporal interaction methods enable identification and characterization of lipids [17,20,21]. Particularly, matrix-assisted laser desorption/ionization mass spectrometry (MALDI-MS) can reveal the distribution of hundreds of ion signals and helps in understanding the cellular profile of the biological system. MALDI-IMS has already revealed the characteristic distribution of several kinds of lipids in various tissues, states or diseases opening a new frontier in the field of lipidomics [162]. Several studies used this method to describe the lipid profile in a variety of cancer types, including CRC [163,164,165,166].

Here, we summarize the latest updates depicting how anabolic or catabolic lipid processes can affect the initiation, progression and metastasis of CRC. Recent studies describe new emerging mechanisms for lipid metabolism supporting CRC growth, such us upregulation of genes for fatty acid synthesis, FA uptake or intracellular lipid accumulation. Another important aspect discussed is the metabolic lipid interplay within the TME. Rewiring of lipid metabolism in immune cells or competition for FAs with cancer cells can lead to immunosuppression and immune evasion of tumors. Moreover, other components of the TME such us CAFs and adipocytes can support the metabolic requirements of tumor cells by secreting FAs, leading to invasion and metastasis. Collectively, these events shape an environment favoring tumor development, but also withhold possible vulnerabilities of tumors for therapeutic intervention, thus adding lipid metabolism as an unquestionable target of CRC.

Nowadays, the treatment options for CRC include surgical removal of tumors, chemotherapy, targeted therapy, radiotherapy and immunotherapy. However, not all patients respond to the available therapeutic approaches, therefore, the discovery of biomarkers and new targets is crucial [167]. Interventions in metabolic pathways can lead to reprogramming of cells and have shown some clinical benefits, on their own or in combination with conventional or immune therapies. A growing number of small-molecule inhibitors tested in many preclinical models strongly support this use and application to interfere with lipid homeostasis and urges the advance of clinical use as of today no lipid-targeted therapies are currently used for CRC [22,79]. 

Knowledge about complex networks of lipid metabolism and function has just started to be untangled. It will be of critical importance in the future to take the complexity of lipid metabolism into consideration but at the same time to draw meaningful conclusions by simplifying complex concepts with targetable pathways or representative biomarkers. The question of timing poses another important feature to be addressed. At what time point and process of cell dissemination is lipid metabolism essentially involved? Spatiotemporal integration, distribution and alignment with gene expression or protein interaction networks still pose as ongoing technical challenge but are future valuable techniques to gain more insights on whole cell and tissues than just single snapshots at one time point.

Although our knowledge is still incomplete, understanding the complex cell communications and lipid metabolic interplay within the CRC tumors in depth offers the possibility of new treatment discovery.

## Abbreviations

ACC	acetyl-CoA-carboxylase
ACLY	ATP-citrate lyase
ACSS	acyl coenzyme A synthetase
ALA	α-linolenic acid
AMPK	5′AMP-activated protein kinase
CACT	carnitine-acylcarnitine-transporter
CAF	cancer associated fibroblast
CE	cholesteryl esters
CLA	conjugated linoleic acid
CPT	carnitine palmitoyltransferase
CRC	colorectal cancer
EC	endothelial cell
EGFR	epidermal growth factor receptor
ELOVL	elongation of very-long-chain fatty acids protein
EPCAM	epithelial cell adhesion molecule
FA	fatty acid
FABP	fatty acid binding protein
FADS	fatty acid desaturase
FAO	fatty acid oxidation
FASN	fatty acid synthase
FAT	fatty acid translocase
FATP	fatty acid transport protein
FFAR-2	free fatty acid receptor-2
HOXC10	homeodomain-vontaining gene C10
IL-4	interleukin-4
LA	linoleic acid
LCFA	long-chain fatty acid
LD	lipid droplet
LDL	low-density lipoprotein
LPS	lipopolysachcharide
LXRs	endothelial liver X receptors
MCFA	medium-chain fatty acid
MUFA	monosaturated fatty acid
NK	natural killer cell
oxLDL	oxidized low density lipoprotein
PMN-MDSC	myeloid-derived suppressor cell
PPAR-γ	peroxisome proliferator-activated receptor-γ
PUFA	polyunsaturated fatty acid
ROS	reactive oxygen species
SCD	stearoyl-CoA desaturase
SCFA	short-chain fatty acid
SFA	saturated fatty acid
TAG	triacylglycerols
TAM	tumor associated macrophage
TCA cycle	tricarboxylic acid cycle
Th17	T Helper 17
TGF-β	transforming growth factor
TME	tumor microenvironment
Treg	T Regulatory
TRM	tissue resident memory
TSP-1	thrombospondin-1
UCP	mitochondrial uncoupling protein
UFA	unsaturated fatty acids
VEGFR-2	vascular endothelial growth factor receptor-2
VLCFA	very long-chain fatty acid
VLDL	very low-density lipoprotein
WAT	white adipose tissue
